# Kif21B mediates the effect of estradiol on the morphological plasticity of mouse hippocampal neurons

**DOI:** 10.3389/fnmol.2023.1143024

**Published:** 2023-04-03

**Authors:** Danny Ganchala, Daniel Pinto-Benito, Elisa Baides, Isabel Ruiz-Palmero, Daniela Grassi, Maria Angeles Arevalo

**Affiliations:** ^1^Instituto Cajal (IC), CSIC, Madrid, Spain; ^2^Centro de Investigación Biomédica en Red de Fragilidad y Envejecimiento Saludable (CIBERFES), Instituto de Salud Carlos III, Madrid, Spain; ^3^Servicio de Proteómica, Instituto Biosanitario de Granada-IBS, Fundación Para la Investigación Biosanitaria de Andalucía Oriental—Alejandro Otero (FIBAO), Antiguo Hospital Universitario San Cecilio, Unidad de Apoyo a la Investigación (UNAI), Granada, Spain; ^4^Department of Anatomy, Histology and Neuroscience, Autonoma University of Madrid, Madrid, Spain

**Keywords:** Kif21B, estradiol, BDNF, neuron morphology, Ngn3

## Abstract

**Introduction:**

Neurons are polarized cells, and their ability to change their morphology has a functional implication in the development and plasticity of the nervous system in order to establish new connections. Extracellular factors strongly influence neuronal shape and connectivity. For instance, the developmental actions of estradiol on hippocampal neurons are well characterized, and we have demonstrated in previous studies that Ngn3 mediates these actions. On the other hand, Kif21B regulates microtubule dynamics and carries out retrograde transport of the TrkB/brain-derived neurotrophic factor (BDNF) complex, essential for neuronal development.

**Methods:**

In the present study, we assessed the involvement of kinesin Kif21B in the estradiol-dependent signaling mechanisms to regulate neuritogenesis through cultured mouse hippocampal neurons.

**Results:**

We show that estradiol treatment increases BDNF expression, and estradiol and BDNF modify neuron morphology through TrkB signaling. Treatment with K252a, a TrkB inhibitor, decreases dendrite branching without affecting axonal length, whereas. Combined with estradiol or BDNF, it blocks their effects on axons but not dendrites. Notably, the downregulation of Kif21B abolishes the actions of estradiol and BDNF in both the axon and dendrites. In addition, Kif21B silencing also decreases Ngn3 expression, and downregulation of Ngn3 blocks the effect of BDNF on neuron morphology.

**Discussion:**

These results suggest that Kif21B is required for the effects of estradiol and BDNF on neuronal morphology, but phosphorylation-mediated activation of TrkB is essential only for axonal growth. Our results show that the Estradiol/BDNF/TrkB/Kif21B/Ngn3 is a new and essential pathway mediating hippocampal neuron development.

## 1. Introduction

Neurons are polarized cells both structurally and functionally. Proneural signaling is activated during development under physiological conditions and reactivated in the adult organism to repair damage in case of injury. For this, modification of the cell morphology is required (regarding the number of dendrites, length, branching, and synaptic input) in order to establish new connections. These morphological changes constitute the cellular and molecular bases of the so-called neuronal plasticity. Dendrite morphology is regarded as a phenotypic property that can be drastically altered by extrinsic factors such as activation of the Notch receptor and neurotrophins (Whitford et al., 2002; Salama-Cohen et al., 2005; Salazar et al., 2020). Notch activity strongly impacts the morphology of both developing and postmitotic neurons. Its activation affects the growth pattern of dendrites by controlling the expression of a series of proneural genes such as neurogenin 3 (Ngn3), which is known to be expressed in the hippocampus from mouse embryos to adult animals, and controls the number of dendrites (Salama-Cohen et al., 2006; Simon-Areces et al., 2010) and synaptogenesis by promoting the expression of Formin1, a crucial component of the Ngn3 signaling pathway (Simon-Areces et al., 2011). In the hypothalamus, Ngn3 promotes Pomc and repress Npy expression, neuropeptides that are critical for the differentiation of hypothalamic neuronal populations regulating food intake and energy homeostasis (Pelling et al., 2011) and we recently showed that it mediates the higher Pomc and lower Npy levels observed in females when compared to male neurons (Cabrera Zapata et al., 2022). Estradiol has previously been shown to increase neuronal branching and neurite length (Audesirk et al., 2003; Carrer et al., 2005; Ruiz-Palmero et al., 2011, 2016) and that these effects are mediated by Ngn3.

There are two mechanisms by which estradiol can act on differentiation patterns and neuronal development. First, by binding to membrane estrogen receptors, estradiol induces phosphorylation and activation of ERK promoting its translocation to the nucleus to regulate genes associated to the cytoskeleton and second, by indirect autocrine and local paracrine mechanisms, which are mediated by growth factors (Singh et al., 1999; Arevalo et al., 2015a). Estradiol is known to regulate brain-derived neurotrophic factor (BDNF) transcription in the nervous system since the gene encoding for this neurotrophin has been reported to contain a sequence similar to the canonical estrogen response element found in estrogen target genes (Sohrabji et al., 1995). BDNF regulates neuron development and thus may participate in the developmental actions of estradiol on neurons. The role of BDNF in regulating neuron morphology and plasticity has been established in a variety of preparations (McAllister et al., 1997; Jin et al., 2003; Bazzari and Bazzari, 2022), and most studies of signaling pathways involved in the effects of neurotrophins on neurite outgrowth emphasize the importance of Trk receptors.

In order to activate signaling pathways triggered by neurotrophins, the neurotrophin-receptor complexes form signaling endosomes, which will be transported retrogradely to the nucleus where transcription takes place. Although it has been proposed that this process occurs through dynein networks, some kinesins, like Kif21B, are also involved in this mechanism. The expression of Kif21B, is restricted to the brain, spleen, and testes (Marszalek et al., 1999) and within neurons, Kif21B is enriched in dendrites compared to soma and axons and in neuronal growth cones (Marszalek et al., 1999; Huang and Banker, 2012; Ghiretti et al., 2016; Muhia et al., 2016). Mutations in the Kif21B kinesin gene provoke neurodevelopmental disorders through imbalanced canonical motor activity (Asselin et al., 2020; Narayanan et al., 2022). In addition Kif21B knockout mouse neurons show decreased dendritic branching and spine density, and these structural changes are reflected functionally in learning and memory deficits (Muhia et al., 2016).

**Figure 1:**
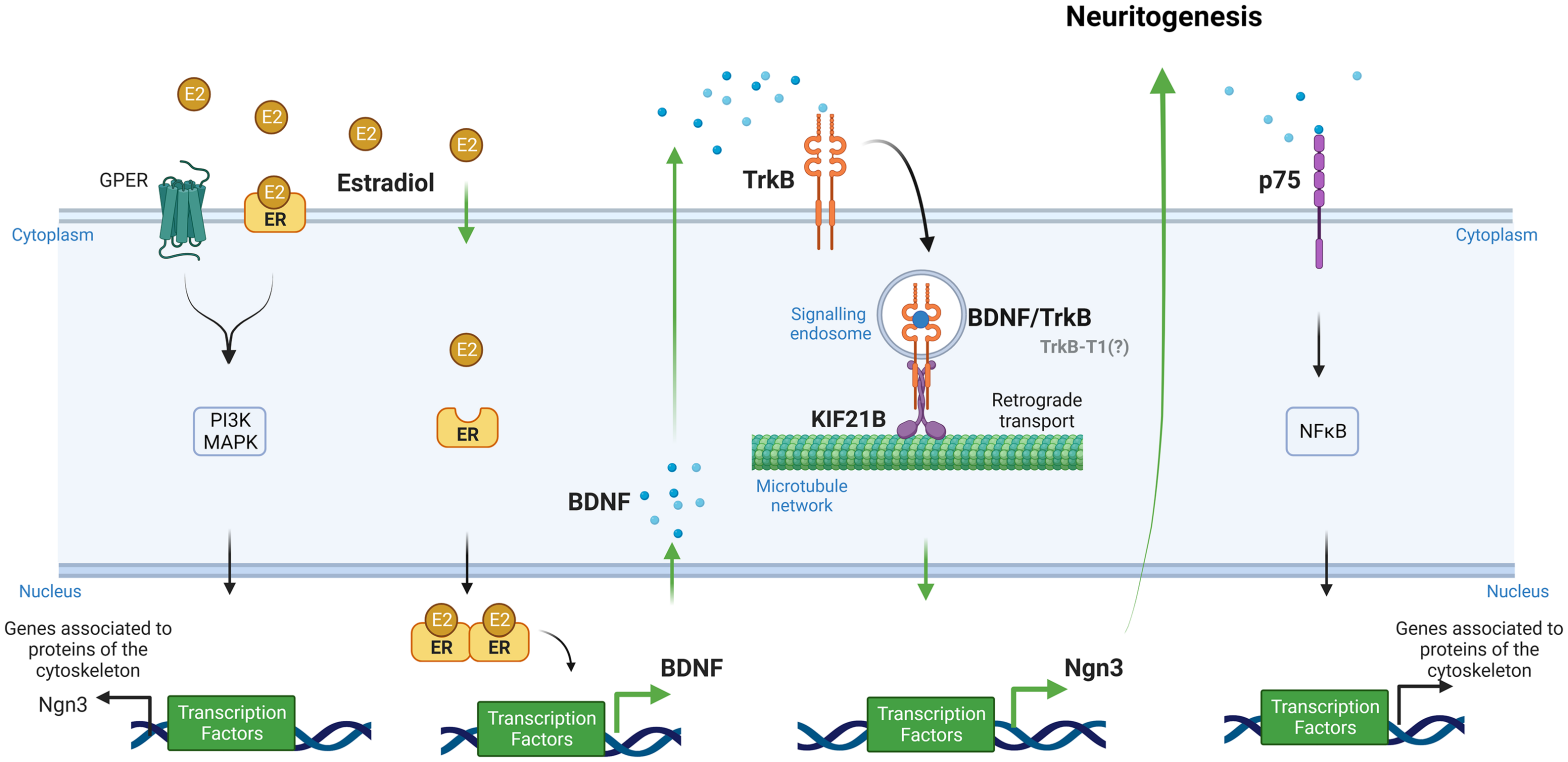
Signaling pathways implicated in the effect of estradiol on the differentiation and plasticity of hippocampal neurons (Created with BioRender.com). Neuroactive steroids and neurotrophins have similar functions in the brain due to their convergence at several points in their signaling pathways. One of these functions is neuritogenesis. Our study proposes a new signaling cascade by which estradiol modifies neuronal morphology. Estradiol increases brain-derived neurotrophic factor (BDNF) mRNA through an estrogen response element (ERE) *via* the canonical pathway. Neurons secrete endogenous BDNF, and this neurotrophin binds to the TrkB receptor forming the BDNF/TrkB complex. Cells endocyte this complex, and the kinesin KIF21B transports it retrogradely along the microtubule network. Ultimately, this leads to the expression of Ngn3, which regulates neuritogenesis. Additionally, estradiol can activate other signaling pathways, namely, PI3K and MAPK, by binding to GPER and membrane receptors, leading to the transcription of genes associated with cytoskeleton proteins. BDNF also can bind to its low-affinity receptor p75, which activates NFκB and regulates molecular cascades that change genes related to cytoskeleton modifications (Green arrows show the steps taken to test the hypothesis proposed by our study to explain the implication of Kif21B in the effect of estradiol. Black arrows show data reported in previous studies).

Evidence has been recently provided for a model in which Kif21B motor teams use microtubules track switching and regulation of microtubule dynamics to traffic cargoes with directional retrograde bias in the neuron dendrites (Masucci et al., 2022). Finally, Ghiretti and colleagues showed that Kif21B contributes to the retrograde trafficking of BDNF–TrkB complexes (Ghiretti et al., 2016). However, the implication of Kif21B in the effects of estradiol on neuron morphology modulation has not been explored so far. In this study, we postulate the involvement of kinesin Kif21B in the estradiol-dependent signaling mechanisms regulating neuritogenesis in primary mouse hippocampal neurons and describe a new signaling pathway by which estradiol is able to modify neuronal morphology involving retrograde transport of BDNF–TrkB through Kif21B (Figure 1).

## 2. Materials and methods

### 2.1. Animals

CD1 mice raised in our in-house colony at the Instituto Cajal (CSIC, Madrid, Spain) were used for this study. Animals received water and food *ad libitum* and were kept in controlled macroenvironmental temperature conditions at 22 ± 2°C and a 12 h light/12 h dark periodic cycle. Procedures for care, welfare, and proper use of all experimental animals followed the European Parliament and Council Directive (2010/63/EU) and the Spanish regulation (R.D. 53/2013 and Ley 6/2013, 11th June) and were approved by our Institutional Animal Care and Use Committee (Comité de Ética de Experimentación Animal del Instituto Cajal) and by the Consejería del Medio Ambiente y Territorio (Comunidad de Madrid, PROEX 134/17).

### 2.2. Hippocampal neuronal cultures and treatment

CD1 mouse embryos at 17 days of gestation (E17, defining E0 as the day of the vaginal plug) were used to establish primary hippocampal neuronal cultures. Pregnant females were sacrificed by cervical dislocation, and embryos were dissected from the uterus. Neurons were cultured separately according to sex by observing the presence/absence of the testis in the male fetuses under a dissecting microscope. The hippocampi were dissected out and stripped off the meninges. Blocks of tissue were incubated for 15 min at 37°C with 0.5% trypsin (Gibco, United States) and then washed three times with Ca^2+^/Mg^2+^-free Hank’s Buffered Salt Solution (Gibco). Finally, tissues were mechanically dissociated into single cells in 37°C warm culture medium, and cells were seeded. The medium was phenol red-free Neurobasal (Gibco) to avoid “estrogen-like effects” (Berthois et al., 1986) and was supplemented with B-27, 0.043% L-alanyl-L-glutamine (GlutaMAX-I) and 1% antibiotic-antimycotic containing 10.000 U/mL penicillin, 10.000 μg/mL streptomycin, and 25 μg/mL amphotericin B (Gibco). Cells were plated on six-well plates (Falcon, United States) at a density of 500–1,000 cells/mm^2^ for mRNA and protein measurements or on 10 mm glass coverslips (Assistent, Germany) at a density of 300–700 cells/mm^2^ for morphological studies. The surfaces of glass coverslips and plates were pre-coated with 500 μg/mL poly-L-lysine (Sigma-Aldrich, United States). After 3 days *in vitro* (3 DIV), some cultures were transfected with siRNAs targeting Kif21B or Ngn3 and/or treated with 2.5 ng/mL (100 nM) BDNF, 10 nM 17β-Estradiol (E2), 200 nM K252a (all from Sigma-Aldrich), or vehicle for 24 h.

### 2.3. Small interfering RNA transfection

Neurons were transfected by electroporation or lipofection using a mixture of four different siRNA sequences targeting Kif21B, or a mixture of two different siRNA sequences targeting *Ngn3* transcripts at a final concentration of 40 nM total RNA (Dharmacon, United States) and a non-targeting siRNA sequence (ntRNA; Dharmacon) was used as control. Co-transfection with pmaxGFP (Lonza, Switzerland) was performed for transfected neuron identification in all cases. According to the manufacturer’s instructions, for immunofluorescence, neurons were transfected by lipofection at 3 DIV with target siRNA or ntRNA using Effectene Transfection Reagent (Qiagen, Germany). For gene expression analysis, neurons were transfected by electroporation before seeding with target siRNA or ntRNA using a 4D-Nucleofector X Unit and the corresponding P3 Primary Cell nucleofection kit (Lonza) according to the manufacturer’s instructions, seeded and incubated for 3 DIV until processing for RNA isolation or western blot.

### 2.4. RNA isolation and reverse transcription quantitative real-time PCR

Total RNA was extracted and purified using the Illustra RNAspin Mini RNA isolation kit (GE Healthcare, Germany) and quantified by spectrophotometry on NanoDrop One (Thermo Fisher Scientific, United States). 1 μg of RNA per sample was reverse transcribed to cDNA in a 20 μL reaction using M-MLV reverse transcriptase (Promega, United States) and random primers (Invitrogen, United States), following manufacturer’s instructions. qPCR reactions were performed on a 7500 Real-Time PCR System (Applied Biosystems, United States) using SYBR Green Universal PCR Master Mix or TaqMan Gene Expression Master Mix (Applied Biosystems). Primers for BDNF, 18S rRNA and Rpl13a (Table 1) were designed using the online Primer-Basic Local Alignment Search Tool (Primer-BLAST; National Institutes of Health, United States), selecting primer pairs spanning an exon-exon junction to restrict amplification specifically to mRNA. Taqman probes and primers for Ngn3 and Kif21B were Assay-on-Demand gene expression products (Applied Biosystems). All primers were verified to amplify with a 95–100% efficiency by performing four-point calibration curves. Real-time PCRs were performed according to the manufacturer’s instructions using the TaqMan or SYBR Green Universal PCR Master Mix. All reactions were done in duplicates from at least four different cultures. Relative quantification of mRNA expression was determined with the ΔΔCt method, using the BestKeeper index (Pfaffl et al., 2004) calculated for each sample from the Ct values of *Rn18s* (18S rRNA) and *Rpl13a* as control housekeeping genes. Control male samples were used as the reference group.

**Table 1:** Primer sequences for real-time PCR.

Primer	Forward 5′–3′	Reverse 3′–5′
*18S rRNA*	CGCCGCTAGAGGTGAAATTCT	CATTCTTGGCAAATGCTTTCG
*Rpl13a*	TACCAGAAAGTTTGCTTACCTGGG	TGCCTGTTTCCGTAACCTCAAG
*Bdnf*	GAAGGCTGCAGGGGCATAGAC	TACACAGGAAGTGTCTATCCTTA

### 2.5. ELISA assay

Neuronal culture supernatants from six-well plates were collected, centrifuged, and stored at −20°C until use. The secreted BDNF was quantified by Colorimetric Sandwich ELISA Kit (Proteintech, United States). Briefly, reagents, standards, and samples were prepared prior use and were run in duplicate. The plate was incubated at 37°C in a humid environment. A standard curve calibration of seven concentration points was prepared, ranging from 12.5 to 800 pg/mL. 100 μL of sample or standard were placed on the microwells; the plate was sealed and incubated for 2 h. After that, the wells were washed four times, and the detection antibody was added. The plate was incubated for 1 h and washed four times. Next, the HRP-conjugated secondary antibody was added, and the plate was incubated for another 40 min and rewashed four times. The signal was developed with tetramethylbenzidine substrate, and the color obtained changed from blue to yellow by adding the stop solution. Measures were taken at 450 nm with a correction wavelength set a 630 nm. The best-fit standard curve was determined by regression analysis.

### 2.6. Western blot

Cells were lysed and solubilized in 200 μL of Laemmli loading buffer. Samples were boiled and sonicated for 5 min. Solubilized proteins (20 μL) were resolved by 15% SDS–PAGE and then electrophoretically transferred to 0.2 μm Trans-Blot Turbo nitrocellulose membranes (BioRad, Alcobendas, Madrid). Membranes were blocked for 90 min at room temperature (RT) in Tris-buffered saline containing 0.1% Tween 20 and 5% BSA and then incubated overnight at 4°C with anti-Ngn3 rabbit polyclonal antibody (Abcam, Cat# ab38548, diluted 1:500) and anti-βIII tubulin mouse monoclonal antibody (TUJ1, dilution 1:1000 Covance, Emeryville, CA, United States) to ensure equal protein loading. Afterward, membranes were incubated for 1 h at RT with 1:10,000 infrared dye-conjugated secondary antibodies (LI-COR, United States), and proteins were visualized by Odyssey Infrared Imaging System (LI-COR). Densitometric analyses were performed using the ImageJ software (National Institutes of Health; freely available at https://imagej.nih.gov). Data are presented as a Ngn3/βIII-tubulin ratio from 3 to 4 independent cultures.

### 2.7. Immunofluorescence

Neurons were fixed for 20 min at room temperature (RT) in 4% paraformaldehyde prewarmed to 37°C, rinsed and permeabilized for 6 min with 0.12% Triton-X plus 0.12% gelatin in phosphate-buffered saline (PBS), blocked 1 h at RT in PBS/gelatin, and incubated for 1 h at RT with anti-microtubule associated protein 2 (MAP2) mouse monoclonal antibody (diluted 1:200 in PBS/gelatin; Sigma-Aldrich) and with anti-Tau rabbit polyclonal antibody (diluted 1:500 in PBS/gelatin; Abcam, United Kingdom). After rinsing with PBS, cells were incubated for 1 h at RT with the secondary antibodies Alexa 594 goat anti-mouse for the detection of MAP2, and Alexa 488 goat anti-rabbit, for the detection of Tau (diluted 1:1,000 in PBS/gelatin; Jackson ImmunoResearch, United States). After washing with PBS, glass coverslips were mounted on slides with Vectashield antifade mounting medium containing DAPI (Vector Laboratories, Burlingame, CA, United States).

### 2.8. Imaging and morphometric analysis

Image analysis of the neuronal cultures was performed on microphotographs obtained on a Leica DMI6000 fluorescent microscope (Leica, Germany) using a 20X objective and a Leica DFC350 FX digital camera. At 4 DIV, the minor processes/dendrites were identified as MAP2 immunoreactive neurites with acute-angled branching. In contrast, the axon was recognized as a single, thinner neurite of homogeneous caliber along its entire length, at least three times longer than the other processes, with right-angled branching and Tau selective immunoreactivity. Evaluation of neuritic arborization was carried out by a Sholl analysis (Sholl, 1953) performed with the CellTarget software (Garcia-Segura and Perez-Marquez, 2014; freely available at https://www3.uah.es/biologia_celular/JPM/CellTarget/CellTarget.html). A grid of six concentric circles with an increasing radius of 5 μm with respect to the previous one was placed centered on the cell soma, and the number of times neurites intersected any circle was counted in about 30 neurons per experimental condition and culture (four independent cultures). The total number of intersections per neuron was obtained and interpreted as arborization complexity. The axonal length was randomly measured in 50–100 neurons per experimental condition and culture (four independent cultures) using the NeuronJ ImageJ plugin (Meijering et al., 2004; freely available at https://imagescience.org/meijering/software/neuronj/).

### 2.9. Statistical analysis

Data are presented as mean ± SEM. Statistical analyses were carried out using GraphPad Prism software version 8.0 for Windows. The normality of the data was assessed with the Kolmogorov–Smirnov test to satisfy the assumption of normality for the ANOVA. Differences between two experimental groups were analyzed by Student’s *t*-test. Interaction between sex and treatment was determined using the two-way ANOVA interaction model, with Tukey’s *post hoc* multiple comparisons. *p* < 0.05 was considered statistically significant. Sample size (*n*) is indicated in the figure legends and was 3–6 independent cultures for RT-qPCR/Western blot experiments or 50–100 neurons from at least four independent cultures for immunofluorescence analysis. The number of independent cultures corresponds to the number of pregnant mothers from which embryos were obtained.

## 3. Results

### 3.1. Estradiol and BDNF equally increase axonal length and dendritic arborization of primary mouse hippocampal neurons

**Figure 2:**
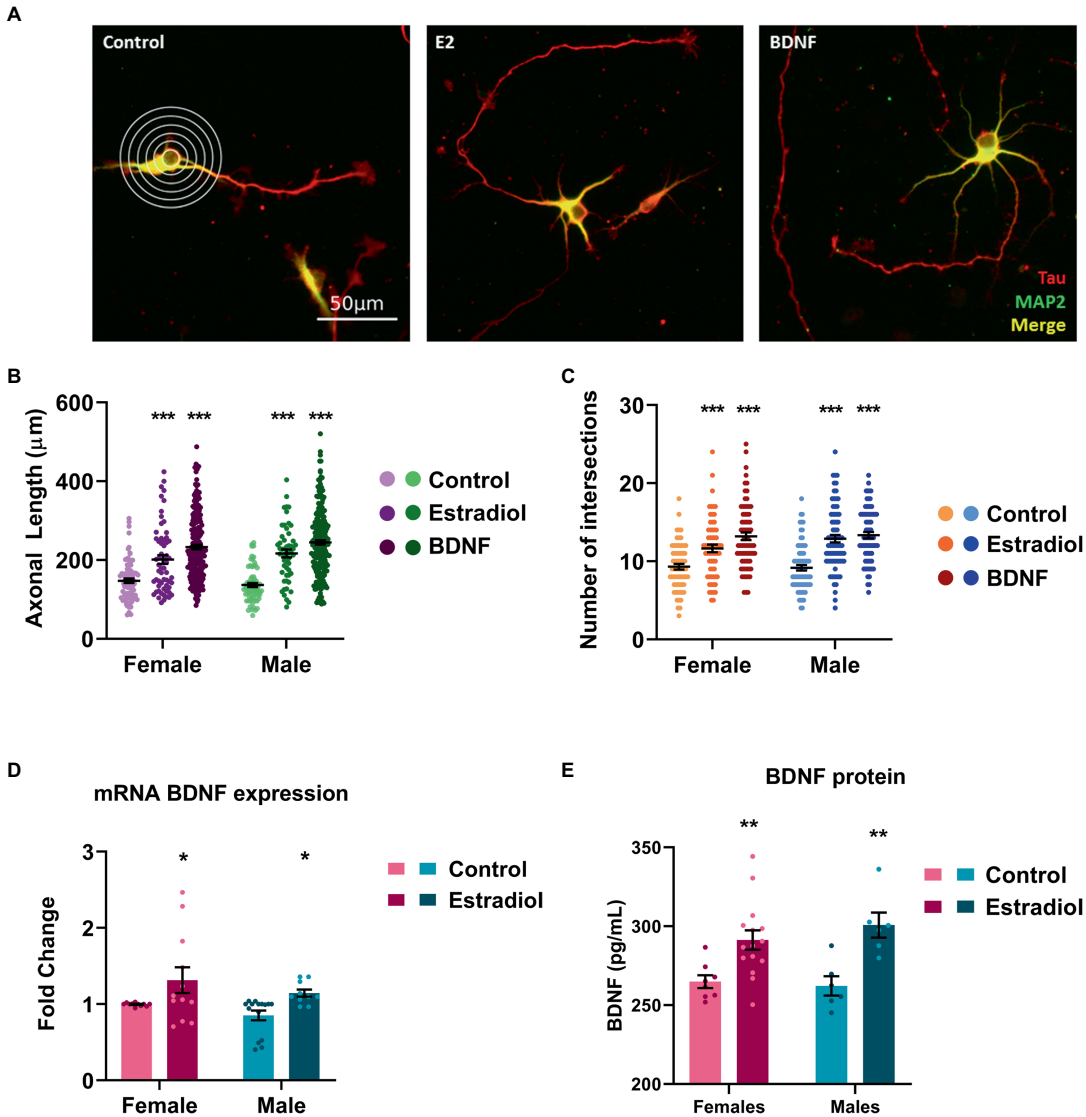
Characterization of the morphological changes induced by exogenous estradiol and BDNF in primary cultures of hippocampal neurons. **(A)** Representative example of four DIV neurons treated with estradiol and BDNF. In the left panel, the grid used for Sholl analysis is shown. Neurons are immunostained for MAP2 (green) and Tau (red). **(B,C)** Quantitative analysis of axonal length and complexity of the dendritic arbor (number of intersections of dendrites with the circles in the Sholl analysis) of sex-separated cultures treated with estradiol and BDNF. Neurons quantified (50–100) are at least from four independent experiments. Data represent the mean ± SEM; two-way ANOVA with Tukey’s multiple comparisons: ^***^p < 0.001 vs. control groups. **(D)** BDNF mRNA levels in male and female neurons treated with estradiol *N* ≥ 4. Data represent the mean ± SEM; two-way ANOVA with Tukey’s multiple comparisons: ^*^p < 0.05 vs. control groups. **(E)** BDNF protein levels in male and female neurons treated with estradiol *N* ≥ 4. Data represent the mean ± SEM; two-way ANOVA with Sidak’s multiple comparisons: ^**^p < 0.01 vs. control groups.

Primary neuronal cultures were treated with estradiol or BDNF at 3 DIV, and, after 24 h, cells were stained with anti-MAP2 antibody to label dendrites (shown in green). In contrast axons were labeled with anti-Tau-1 antibody (shown in red; Figure 2A). Both treatments significantly increased the axonal length (Figure 2B) and the number of intersections of the processes with the lines of the grid used in Sholl analysis (Figure 2C). However, no sex differences either in controls or in treated cells have been detected in both the evaluated parameters.

It is known that estradiol is able to increase BDNF levels in several models (Sohrabji et al., 1995; Arevalo et al., 2015b; Shen et al., 2017). Thus, we explored if exogenous estradiol exposure could be able to increase BDNF levels in primary hippocampal neuronal cultures from male and female mice. Results confirmed that in our model, treatment with estradiol also enhances BDNF mRNA and protein expression in both sexes (Figures 2D, E), suggesting that the effect observed on the neuron morphology upon estradiol treatment could be mediated by BDNF signaling.

### 3.2. TrkB tyrosine kinase activity is involved in the effects of estradiol and BDNF on axonal length but not on neuronal dendrites

**Figure 3:**
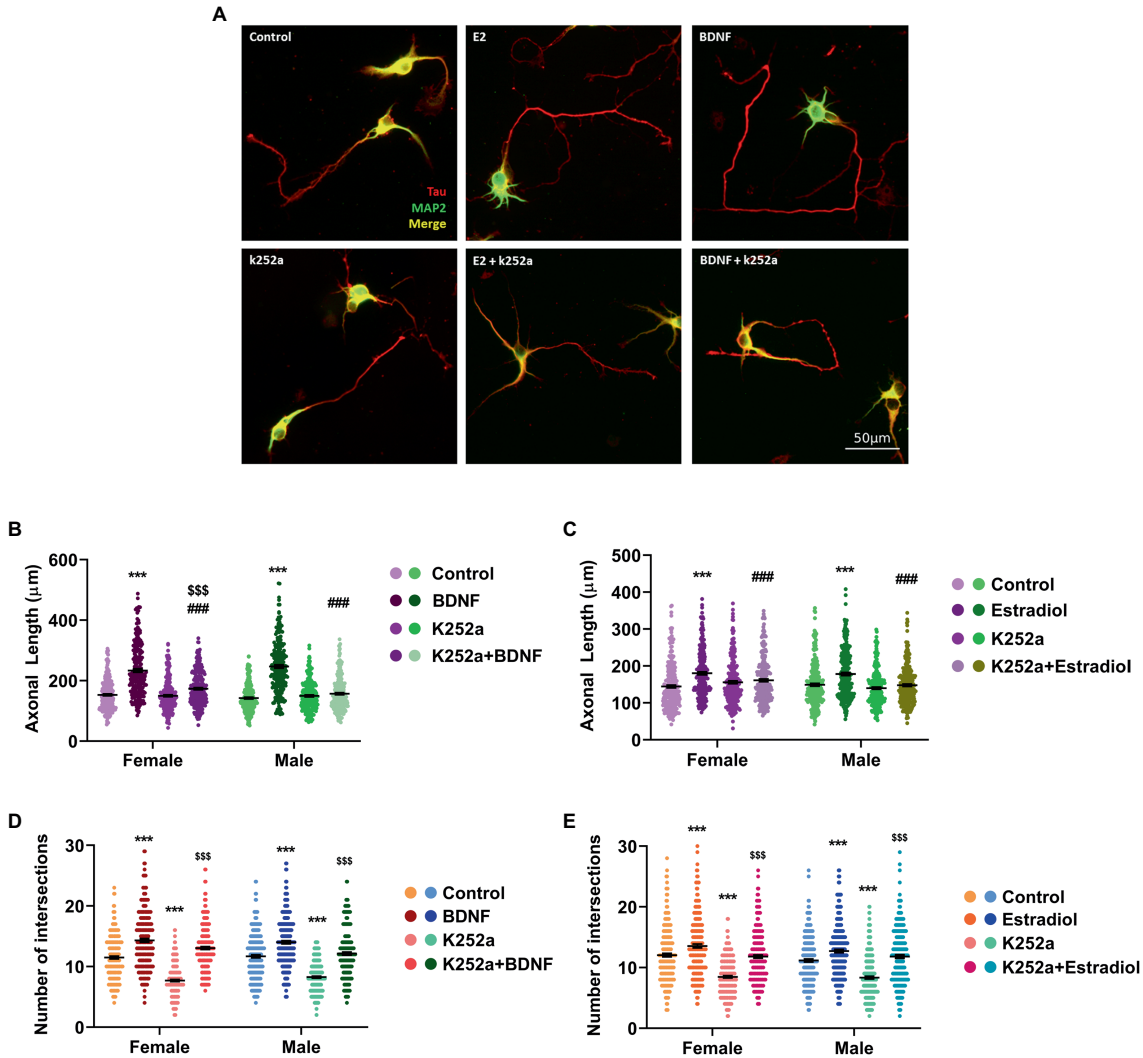
Implication of TrkB receptor in the actions of estradiol and BDNF on neuron morphology. **(A)** Representative example of neurons treated with E2, BDNF, k252a, and the combinations E2 + K252a, BDNF + K252a. Neurons are immunostained for MAP2 (green) and Tau (red). **(B–E)** Quantitative analysis of axonal length and complexity of the dendritic arbor (number of intersections of dendrites with the circles in the Sholl analysis) of sex-separated cultures treated with E2, BDNF, and K252a and the combinations E2 + K252a, BDNF + K252a. Neurons quantified (50–100) are at least from four independent experiments. Data represent the mean ± SEM; two-way ANOVA with Tukey’s multiple comparisons: ^***^p < 0.001 vs. control groups; ^###^*p* < 0.001 vs. BDNF or Estradiol treated groups; and ^$$$^*p* < 0.001 vs. K252a treated groups.

We further assessed if TrkB, the BDNF-specific receptor, could be involved in the neuritogenic action triggered by estradiol. Thus, male and female hippocampal neuronal cultures were treated with K252a, a TrkB inhibitor. Results showed that K252a does not affect the axonal length (Figures 3A–C) but when cells were co-treated in combination with estradiol or BDNF, K252a blocked the increase of axonal length. These results suggest that TrkB activation is necessary for BDNF to exert its effect on axonal elongation (Figure 3B) and that the estradiol action is mediated by the TrkB activation (Figure 3C). Regarding dendrites, K252a decreases the number of intersections in neurons, but the combined treatments with estradiol or BDNF are able to increase the number of intersections reaching control levels (Figures 3D,E). These results indicate that the effect of BDNF on dendritic tree development does not depend exclusively on TrkB phosphorylation (Figure 3D). Estradiol capacity to increase the number of intersections of neurons co-treated with K252a (Figure 3E) suggests that other signaling pathways are mediating this effect even independent to TrkB/BDNF.

### 3.3. Kif21B (the TrkB/BDNF transporter) is involved in the neuritogenic effect of estradiol

TrkB activation is involved in the axogenic action of estradiol in hypothalamic neurons in culture (Brito et al., 2004) and, it has been demonstrated that Kif21B, the kinesin that transports the cargo formed by BDNF/TrkB, is essential for the development of dendritic arbors (Muhia et al., 2016). Based on this evidence, we evaluated the role of Kif21B in the estradiol-triggering neuritogenesis. Thus, we knocked-down Kif21B expression by using siRNAs targeting Kif21B gene, and we exposed the cells to exogenous estradiol and assessed neuronal development.

**Figure 4:**
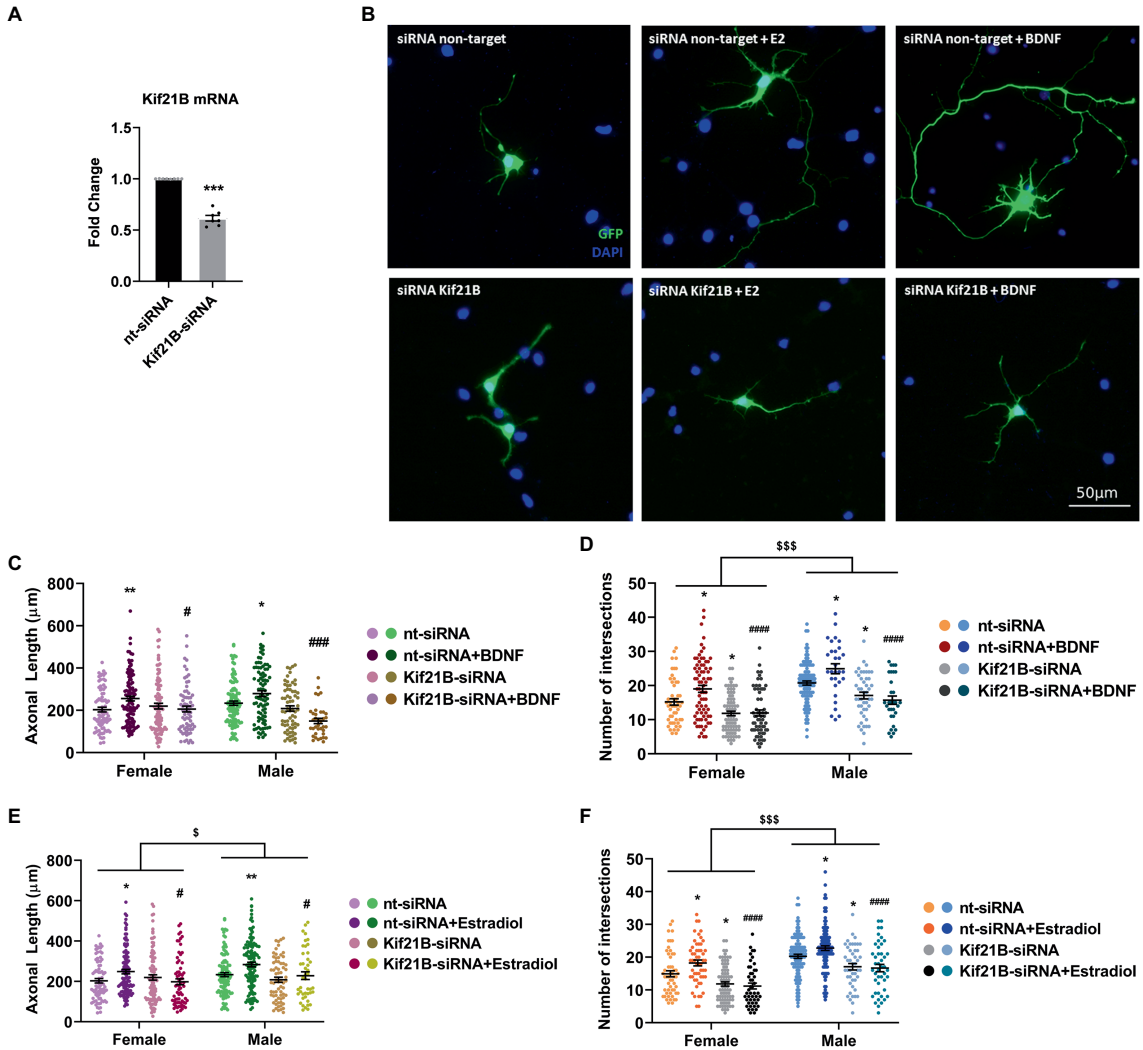
Kif21B silencing impairs the neuritogenic effect of Estradiol and BDNF. **(A)** Downregulation of Kif21B mRNA levels in electroporated cultures with a mix of four specific siRNAs for this gene *N* = 4. Graph represents the mean ± SEM; Unpaired *t*-test: ^***^p < 0.001. **(B)** Representative example of neurons immunostained for Green Fluorescent Protein (GFP, green) and DAPI (blue). Electroporated neurons with non-target siRNA are shown in the panel above and neurons targeted with Kif21B siRNA are displayed in the panel below. **(C–F)** Quantitative analysis of axonal length and complexity of the dendritic arbor (number of intersections with the circles in the Sholl analysis) of sex-separated cultures at 3 DIV with non-target or Kif21B siRNA. Neurons quantified (50–100) are at least from four independent experiments. Data represent the mean ± SEM; two-way ANOVA with Tukey’s multiple comparisons: ^*^p < 0.05, ^**^p < 0.01 vs. nt-siRNA groups; ^#^*p* < 0.05, ^###^*p* < 0.001, ^####^*p* < 0.0001 vs. nt-siRNA + Estradiol or BDNF; ^$^*p* < 0.05, ^$$$^*p* < 0.001 sex differences.

We primarily tested the efficiency of Kif21B siRNA by measuring the levels of Kif21B mRNA in cultures electroporated with specific siRNA targeting this gene. Results showed that Kif21B-siRNA is able to downregulate the gene expression compared to non-target siRNA (Figure 4A).

Subsequently, neurons were co-transfected with pEGFP plus Kif21B-siRNA or non-target siRNA and treated with estradiol or BDNF. Morphological analysis showed that downregulation of Kif21B impairs the effects of exogenous estradiol and BDNF on both axonal length and number of intersections (Figures 4B–F) indicating that the BDNF/TrkB cargo transport kinesin Kif21B is important for BDNF actions not only on axonal growth (Figure 4C) but also on the increase of the number of intersections (Figure 4D) in a sex-different manner. In addition, these results highlighted that the transport of TrkB/BDNF by Kif21B contributes to the estradiol effects on axonal length (Figure 4E) and number of intersections (Figure 4F) and showing sexual differences on both parameters. These results suggest that Kif21B is required for estradiol and BDNF effects on neuronal morphology.

### 3.4. Estradiol and BDNF increase the mRNA expression of Ngn3 through TrkB receptor

**Figure 5:**
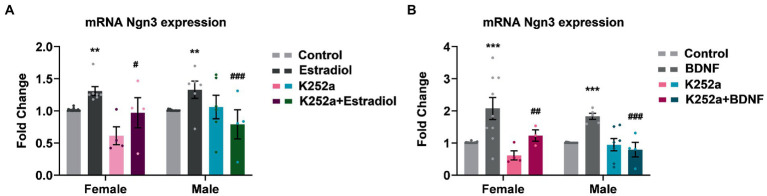
Inhibition of TrkB receptor abrogates the effects of estradiol and BDNF on Ngn3 mRNA expression in neurons. **(A)** Ngn3 mRNA levels in male and female neurons treated with E2, K252a and E2 + K252a. **(B)** Ngn3 mRNA levels in male and female neurons treated with BDNF, K252a and BDNF + K252a; *N* = 4. Graphs represent the mean ± SEM; two-way ANOVA with Tukey’s multiple comparisons: ^**^p < 0.01, ^***^p < 0.001 vs. control groups; ^#^*p* < 0.05, ^##^*p* < 0.01, and ^###^*p* < 0.001 vs. Estradiol or BDNF treated groups.

Since estradiol and BDNF modify neuron morphology *in vitro*, we decided to explore changes in the expression of genes involved in neuritogenesis such as Ngn3, a transcription factor that is involved in the development of hippocampal neurons. We have indeed previously described that estradiol increases Ngn3 expression in cultured hippocampal neurons through PI3K signaling, a TrkB downstream pathway (Ruiz-Palmero et al., 2013). This might be because estradiol and BDNF share similar molecular mechanisms within their signaling pathways, but also due to estradiol modifying the expression of BDNF. To assess if TrkB activation by BDNF is implicated in the enhanced Ngn3 expression induced by estradiol, cultures were co-treated with K252a and estradiol or BDNF. As expected, estradiol treatment increases the mRNA expression of Ngn3. However, estradiol is not able to increase Ngn3 levels in cells co-treated with K252a (Figure 5A). Exogenous BDNF also induced an increase of Ngn3 mRNA expression in cultured neurons and this effect was blocked by K252a (Figure 5B).

**Figure 6:**
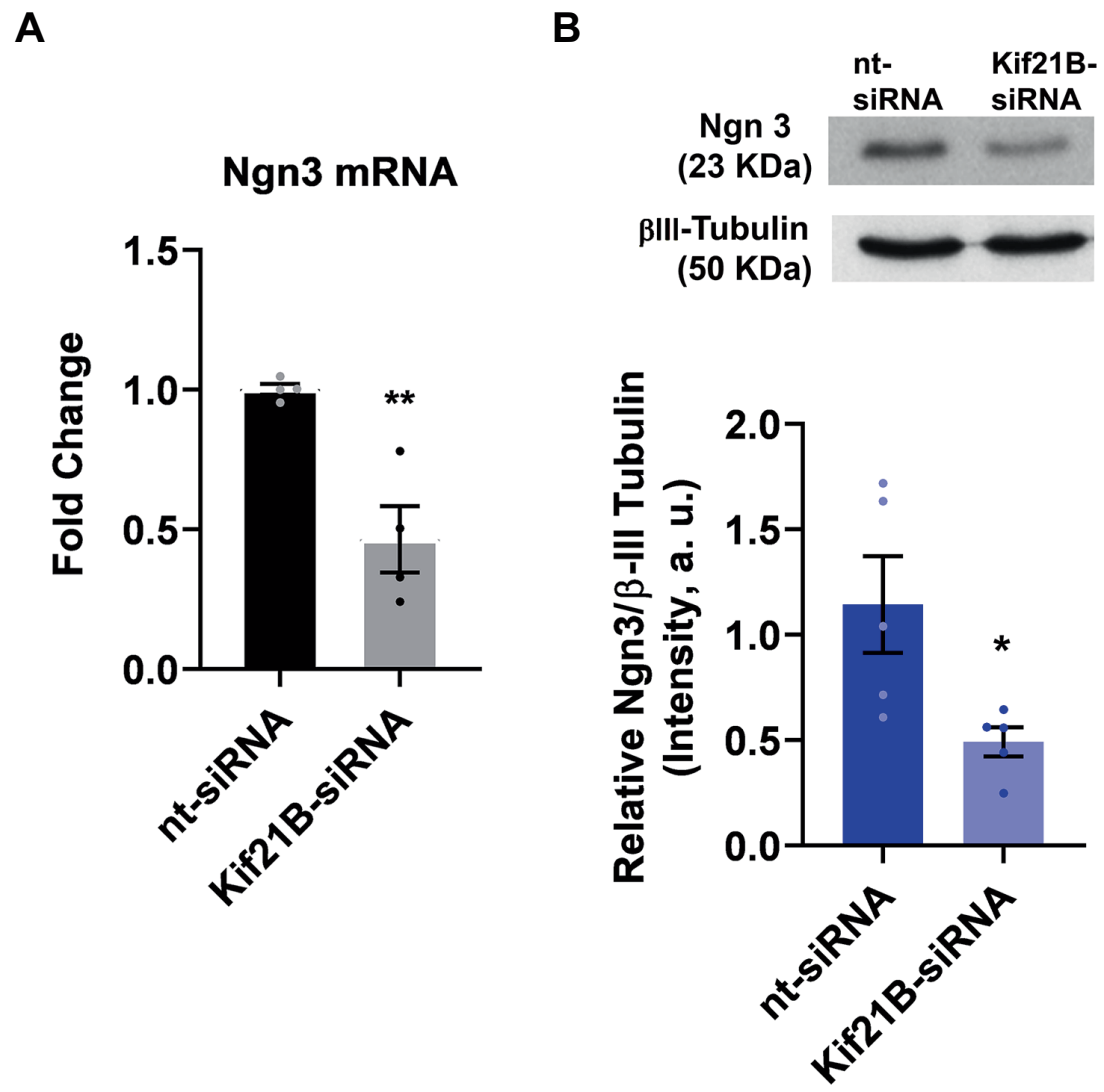
Kif21B silencing downregulates Ngn3 expression. **(A)** Kif21B-siRNA reduces Ngn3 mRNA levels in hippocampal neuron cultures; *N* = 4. **(B)** Ngn3 protein levels in electroporated cultures with Kif21B-siRNA, evaluated by western blot using βIII-tubulin as a loading control; *N* = 5. Graphs represent quantitative analysis expressed as mean ± SEM; Unpaired *t*-test: ^*^p < 0.05, ^**^p < 0.01 vs. nt-siRNA group.

Next, we evaluated if TrkB/BDNF transport by Kif21B is necessary to provoke changes in Ngn3 expression. Data showed that downregulation of Kif21B induces a decrease in Ngn3 mRNA (Figure 6A) and protein levels (Figure 6B). Taken together, these results lead us to infer that the increase in the Ngn3 mRNA expression induced by estradiol and BDNF might be through TrkB receptor activation by BDNF and retrograde transport of the TrKB/BDNF complex by Kif21B.

### 3.5. Knocking-down Ngn3 blocks the BDNF effect on the hippocampal neuron development in cultures

**Figure 7:**
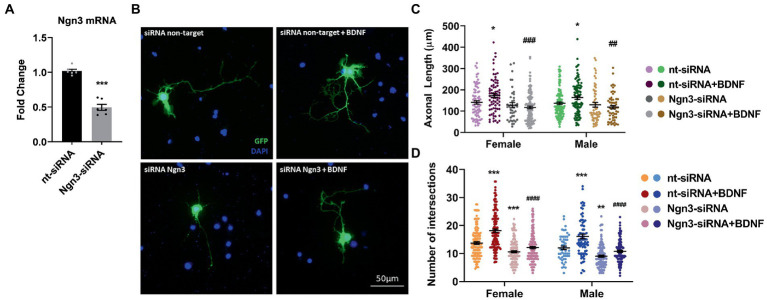
Knocking-down Ngn3 blocks the BDNF effect on the hippocampal neuron development in cultures. **(A)** Downregulation of Ngn3 mRNA levels in electroporated cultures with two specific siRNAs for this gene; *N* = 5. Graphs represent the mean ± SEM; Unpaired *t*-test: ^***^p < 0.001. **(B)** Representative example of neurons immunostained with Green Fluorescent Protein (GFP, green) and DAPI (blue). Electroporated neurons with non-target siRNA are shown in the panel above and neurons targeted with Ngn3 siRNA are displayed in the panel below. **(C)** Quantitative analysis of axonal length and **(D)** complexity of the dendritic arbor (number of intersections of dendrites with the circles in the Sholl analysis) of sexed cultures at 3 DIV with non-target or Ngn3 siRNA. Neurons quantified (50–100) are at least from four independent experiments. Data represent the mean ± SEM; two-way ANOVA with Tukey’s multiple comparisons: ^*^p < 0.05, ^**^p < 0.01, and ^***^p < 0.001 vs. nt-siRNA group; ^##^*p* < 0.01, ^###^*p* < 0.001, and ^####^*p* < 0.0001 vs. nt-siRNA + BDNF group.

In a previous study (Ruiz-Palmero et al., 2011), we showed that Ngn3 signaling is involved in the neuritogenic actions of estradiol on hippocampal neuron morphology. Here we evaluated if Ngn3 is also implicated in the BDNF effects on neuron morphology. We silenced neuron cultures with Ngn3 siRNA to test this hypothesis and treated them with BDNF. First, we electroporated the cells with a mix of two siRNA targeting Ngn3 and we observed a significantly reduced Ngn3 mRNA expression compared to the control siRNA (Figure 7A). Next, we added BDNF to cultures co-transfected with pEGFP-max plus either control siRNA or specific siRNAs and we analyzed the morphology of hippocampal neurons (Figure 7B). Results showed that Ngn3 downregulation impairs the effect of BDNF on axonal length (Figure 7C). In addition, Ngn3 knock-down reduced the complexity of the dendritic arbor by decreasing the number of intersections and blocked the neuritogenic effect of BDNF on neuronal development (Figure 7D).

## 4. Discussion

The present study aimed to find out if Kif21B is involved in the effects of estradiol on the development of hippocampal neurons. First, we analyzed the effects of exogenous estradiol and BDNF on dendritic tree complexity and axonal length on cultures from male and female mice. We found that both treatments modified the morphology of primary mouse hippocampal neurons in a similar way which is in line with previous data from our lab (Singh et al., 2006; Ruiz-Palmero et al., 2011, 2013, 2016) and other authors (Martín-Rodríguez et al., 2020). However, we did not find differences between the sexes. In previous studies we showed that cultured hippocampal neurons present sexual differences due to the endogenous production of estradiol, making female neurons develop faster than male neurons until 3 DIV. Thus, at 2 DIV exogenous estradiol promotes neuritogenesis in male neurons but not in female neurons. These basal sex differences in hippocampal neuron development are associated with sex differences in Ngn3 expression and explain why adding exogenous estradiol to 2 DIV cultures increases the Ngn3 expression only in males. From 3 DIV, Ngn3 basal levels decrease and are similar in male and female neurons (Ruiz-Palmero et al., 2016). Kif21B supports the retrograde trafficking of BDNF–TrkB complexes in dendrites, and for this task, dendrites have to be well specified. Thus, we decided to conduct experiments with 4 DIV cultured neurons since, at this developmental stage, the low expression of Ngn3 can increase by estradiol treatment in both sexes and consequently enhance neuronal development without sexual differences.

Exogenous BDNF treatment induced changes in the hippocampal neuronal morphology that are similar to those induced by estradiol. Actions of estradiol on neuron morphology could be *via* initiation of the mitogen activated protein kinase (MAPK) cascade, which mediates the axogenic effect of estradiol through ERα in hypothalamic neurons (Gorosito and Cambiasso, 2008; Cabrera Zapata et al., 2019). However, in previous work, we revealed that classical estrogen receptors do not participate in the development of hippocampal neurons, but that estradiol modifies neuronal morphology by acting on GPER through a mechanism that involves activation of PI3K signaling and expression of Ngn3 (Ruiz-Palmero et al., 2011, 2013). A later study identified GPER as a neurotrophic promotor for neurite outgrowth of rat E18 hippocampal, but not cortical neurons, indicating that different molecular pathways may account for the neuritogenic effect of estradiol in different brain regions (Pemberton et al., 2022). Similar results are observed with BDNF since it has been shown that during postnatal development BDNF regulates neuronal architecture and spine morphology of neurons within specific brain areas but not others (Zagrebelsky et al., 2018). PI3K activation is downstream of TrkB, the specific receptor of BDNF, and it is known that estradiol increase BDNF levels in several models through the activation of an estrogen response element (ERE) on the BDNF gene *in vivo* (Sohrabji et al., 1995; Arevalo et al., 2015b) and *in vitro* (Shen et al., 2017). Consistent with these observations, our current results show that estradiol can increase BDNF expression in both sexes in cultured hippocampal neurons, so the action of estradiol on neuron development could be mediated in part through BDNF regulation.

The neuritogenic effect of BDNF seems to be regulated through the TrkB receptor, which is transported to dendritic filopodia and distal dendrites, where it can be located in lipid rafts (Suzuki et al., 2004; Cohen-Cory et al., 2010). A gradual increase in BDNF dimerizes TrkB in a dose-dependent manner and initiates signaling pathways such as PI3K. By neurotrophin signaling this pathway regulates Rho, Rac1, and Cdc42 action (Cohen-Cory et al., 2010), involving BDNF in cytoskeleton modifications and therefore, in neuron morphology. Our results here demonstrate that activation of TrkB is necessary for the increase of axonal length by BDNF and also for the estradiol effect. On the contrary, the inhibitor of tyrosine kinase, K252a, decreased the dendritic arborization, but effects of BDNF and estradiol were insensitive to K252a, indicating that kinase activity of TrkB is contributing to dendritic tree development. However, the ability of BDNF and estradiol to increase dendritic complexity also involves additional signaling pathways. A study made in pancreas showed a positive regulation of Ngn3 protein by TrkB. It also described that this regulation is mediated by TrkB-T1, the isoform lacking the intracellular kinase and autophosphorylation domains and consequently its function is independent of the tyrosine kinase activity of TrkB (Shamblott et al., 2016). This alternative pathway could explain why K252a does not block the effect of estradiol and BDNF on neuronal dendrite branching. In addition, we could also consider the activation of the p75 neurotrophin receptor (p75NTR) by BDNF (Tomellini et al., 2014). Although it was published that p75 negatively regulated dendritic complexity (Zagrebelsky et al., 2005), we observed that activation of p75 decreases the number of primary dendrites and increases dendrite length in cultured hippocampal neurons and this effect is mediated by nuclear translocation of NF-kappaB (Salama-Cohen et al., 2005). Furthermore, a recent work confirmed our results and showed that the absence of p75 resulted in a decrease in dendritic arborization of cortical neurons in microfluidic devices (Moya-Alvarado et al., 2023) suggesting that p75 participates in the regulation of dendritic arborization.

Due to evidence that Kif21B motor activity is required for efficient retrograde transport of functional BDNF/TrkB complex to the nucleus (Ghiretti et al., 2016), we decided to study if Kif21B silencing impairs the neuritogenic effect of estradiol and BDNF. As expected, using a siRNA to decrease Kif21B expression, we observed a reduction in the complexity of the dendritic tree in cultured hippocampal neurons with no effect on axonal length. These results suggest a direct impact of Kif21B on the dynamics of microtubules in dendrites independent of the cargo transport function of kinesin and are consistent with studies where neurons from a Kif21B knockdown mouse had alterations in the dendritic tree, with less branches and fewer synaptic spines (Muhia et al., 2016). In addition, the downregulation of Kif21B expression blocked the action of BDNF and estradiol on axons and dendrites. In the above experiments, the number of intersections was identical in untreated controls of both sexes, however, here the number of intersections is higher in nt-siRNA-treated males (control) than in nt-siRNA-treated females (control). We could speculate that female neurons are more sensitive than male ones to the deleterious effects of the transfection process, but we would need more experiments to confirm this. Still, the results show an altered number of intersections when hippocampal neurons of both sexes are silenced with Kif21B siRNA. Together our results suggest that Kif21B is required for estradiol and BDNF to exert their effects on neuronal morphology, but phosphorylation-mediated activation of TrkB is only essential for the actions on axonal growth. The TrkB/BDNF complex is the only cargo transported by Kif21B that is documented (Ghiretti et al., 2016) but it cannot be excluded that the complex be composed by full length TrkB or TrkB-T1.

We also highlight here that the downregulation of Kif21B drives Ngn3 mRNA and protein levels to decrease. To deepen into the signaling that we describe here, we downregulated the expression of Ngn3 in neuronal cultures and observed that in these conditions, additions of BDNF had no effect. Similar outcomes were previously seen with estradiol (Ruiz-Palmero et al., 2011), reinforcing our inference that the effects of estradiol on neuronal morphology are mediated, at least in part, by increased BDNF expression.

**Figure 8:**
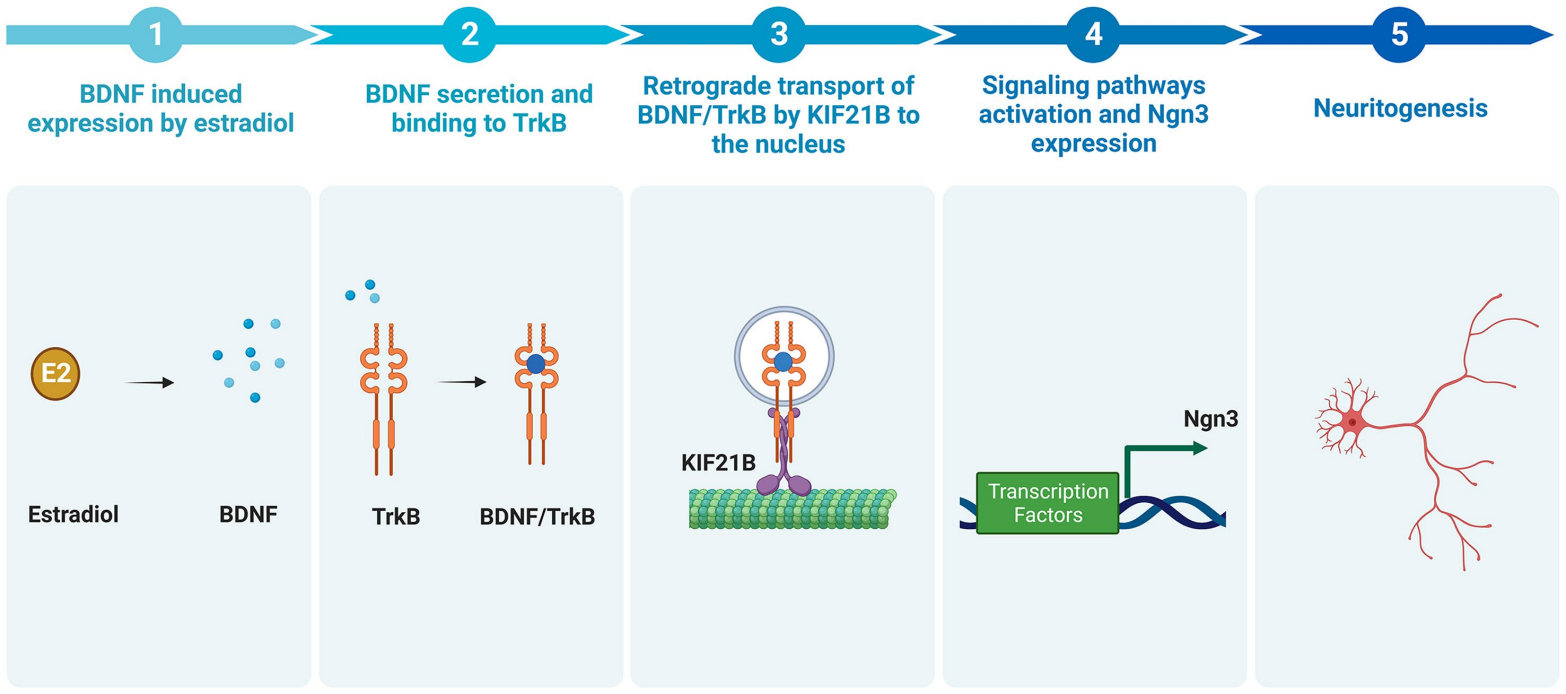
Summary of the newly proposed pathway by which estradiol increases neuritogenesis (Created with BioRender.com). Estradiol increases the expression of BDNF in hippocampal neuronal cultures. BDNF binds to the TrkB receptor. The complex BDNF/TrkB is endocytosed and transported retrogradely by kinesin KIF21B to the soma leading to the activation of signaling pathways, which enhances Ngn3 mRNA and protein level, thus increasing neuritogenesis.

In summary, this work characterizes for the first time a new signaling pathway mediating the development of hippocampal neurons (Figure 8). The data reported here demonstrate that estradiol is able to induce an increase in the BDNF mRNA expression; BDNF binds to the TrkB receptor and the complex is transported in a retrograde way by Kif21B through dendrites to the soma, where it regulates the level of Ngn3 protein *via* TrkB signaling or by affecting gene transcription in the nucleus. This signaling pathway controls the elongation and branching of both axons and dendrites, which determine neuronal shape during development and enable plasticity during adult life.

## Data availability statement

The original contributions presented in the study are included in the article/supplementary material, further inquiries can be directed to the corresponding author.

## Ethics statement

The animal study was reviewed and approved by Comité de Ética de Experimentación Animal del Instituto Cajal (CEEA-IC) and Consejería del Medio Ambiente y Territorio (Comunidad de Madrid, PROEX 134/17).

## Author contributions

MAA conceived and designed the research. DGa, DP-B, EB, and IR-P performed experiments. DGa and DGr analyzed data. DGa elaborated figures and wrote the first draft of the manuscript. DGa, DP-B, EB, IR-P, DGr, and MAA interpreted the results of experiments. MAA edited and revised the manuscript. All authors contributed to the article and approved the submitted version.

## Funding

This study was supported by grants PID2020-115019RB-I00 from Agencia Estatal de Investigación (AEI), Spain, co-funded by Fondo Europeo de Desarrollo Regional (FEDER), SI3-PJI-2021-00508 from Universidad Autónoma de Madrid–Comunidad Autónoma de Madrid, Programa de Estímulo a la Investigación de Jóvenes Doctores, and Centro de Investigación Biomédica en Red de Fragilidad y Envejecimiento Saludable (CIBERFES), Instituto de Salud Carlos III, Madrid, Spain.

## Conflict of interest

The authors declare that the research was conducted in the absence of any commercial or financial relationships that could be construed as a potential conflict of interest.

## Publisher’s note

All claims expressed in this article are solely those of the authors and do not necessarily represent those of their affiliated organizations, or those of the publisher, the editors and the reviewers. Any product that may be evaluated in this article, or claim that may be made by its manufacturer, is not guaranteed or endorsed by the publisher.
